# Prothrombotic autoantibodies in serum from patients hospitalized with COVID-19

**DOI:** 10.1126/scitranslmed.abd3876

**Published:** 2020-11-18

**Authors:** Yu Zuo, Shanea K. Estes, Ramadan A. Ali, Alex A. Gandhi, Srilakshmi Yalavarthi, Hui Shi, Gautam Sule, Kelsey Gockman, Jacqueline A. Madison, Melanie Zuo, Vinita Yadav, Jintao Wang, Wrenn Woodard, Sean P. Lezak, Njira L. Lugogo, Stephanie A. Smith, James H. Morrissey, Yogendra Kanthi, Jason S. Knight

**Affiliations:** 1 Division of Rheumatology, Department of Internal Medicine, University of Michigan, Ann Arbor, MI 48109, USA.; 2 Division of Rheumatology, Ruijin Hospital, Shanghai Jiao Tong University School of Medicine, Shanghai, China.; 3 Division of Geriatric and Palliative Medicine, Department of Internal Medicine, University of Michigan, Ann Arbor, MI 48109, USA.; 4 Division of Cardiovascular Medicine, Department of Internal Medicine, University of Michigan, Ann Arbor, MI 48109, USA.; 5 Division of Intramural Research, National Heart, Lung and Blood Institute, Bethesda, MD 20892, USA.; 6 Michigan Clinical Research Unit, University of Michigan, Ann Arbor, MI 48109, USA.; 7 Division of Pulmonary and Critical Care Medicine, Department of Internal Medicine, University of Michigan, Ann Arbor, MI 48109, USA.; 8 Department of Biological Chemistry, University of Michigan, Ann Arbor, MI 48109, USA.

## Abstract

Patients with COVID-19 are at high risk for thrombotic arterial and venous occlusions. Lung histopathology often reveals fibrin-based blockages in the small blood vessels of patients who succumb to the disease. Antiphospholipid syndrome is an acquired and potentially life-threatening thrombophilia in which patients develop pathogenic autoantibodies targeting phospholipids and phospholipid-binding proteins (aPL antibodies). Case series have recently detected aPL antibodies in patients with COVID-19. Here, we measured eight types of aPL antibodies in serum samples from 172 patients hospitalized with COVID-19. These aPL antibodies included anticardiolipin IgG, IgM, and IgA; anti–β_2_ glycoprotein I IgG, IgM, and IgA; and anti-phosphatidylserine/prothrombin (aPS/PT) IgG and IgM. We detected aPS/PT IgG in 24% of serum samples, anticardiolipin IgM in 23% of samples, and aPS/PT IgM in 18% of samples. Antiphospholipid autoantibodies were present in 52% of serum samples using the manufacturer’s threshold and in 30% using a more stringent cutoff (≥40 ELISA-specific units). Higher titers of aPL antibodies were associated with neutrophil hyperactivity, including the release of neutrophil extracellular traps (NETs), higher platelet counts, more severe respiratory disease, and lower clinical estimated glomerular filtration rate. Similar to IgG from patients with antiphospholipid syndrome, IgG fractions isolated from patients with COVID-19 promoted NET release from neutrophils isolated from healthy individuals. Furthermore, injection of IgG purified from COVID-19 patient serum into mice accelerated venous thrombosis in two mouse models. These findings suggest that half of patients hospitalized with COVID-19 become at least transiently positive for aPL antibodies and that these autoantibodies are potentially pathogenic.

## Casting an autoantibody NET in COVID-19

Patients with severe COVID-19 are at high risk for occlusion of blood vessels of all sizes. This prothrombotic phenotype is reminiscent of patients with lupus and antiphospholipid syndrome, who have long-lived circulating antiphospholipid autoantibodies. In new work, Zuo *et al.* measured eight types of antiphospholipid antibodies in serum from patients hospitalized with COVID-19 and found at least one antibody in half of patients. Antibody levels were associated with neutrophil and coagulation pathway activation. Purified antibodies from some patients activated neutrophils in vitro and potentiated thrombosis when injected into mice. Together, these findings suggest that autoantibodies are a potential therapeutic target in severe COVID-19.

## Teaser

Some patients with severe COVID-19 develop prothrombotic autoantibodies that are similar to antiphospholipid antibodies found in autoimmune diseases.

## INTRODUCTION

Abnormal coagulation characteristics correlate with coronavirus disease 2019 (COVID-19) severity (*1*, *2*). The presence of high D-dimer concentrations in plasma is an independent risk factor for death (*1*, *3*–*5*). Early descriptions of COVID-19 coagulopathy identified this disorder as disseminated intravascular coagulation. However, most patients maintain normal concentrations of coagulation factors, fibrinogen, and platelets, suggesting that COVID-19 induces a unique prothrombotic state that is distinct from traditional descriptions of sepsis-induced coagulopathy (*6*, *7*). There are now increasing reports of venous thromboembolism in patients with COVID-19 (*8*, *9*). This observation is despite concerns regarding underdiagnosis given baseline elevations in the biomarker D-dimer, as well as pragmatic challenges in obtaining diagnostic imaging while patients are in isolation. Arterial thromboses including strokes and myocardial infarctions have also been described (*9*, *10*). Histopathology of lung specimens from patients with severe disease shows not only characteristic findings of acute respiratory distress syndrome (ARDS) but also evidence of fibrin-based occlusion of small blood vessels (*11*–*13*). There are several possible synergistic mechanisms by which severe acute respiratory syndrome coronavirus 2 (SARS-CoV-2) infection may result in macrovascular and microvascular thrombosis (*14*). These include a cytokine storm that activates leukocytes, endothelium, and platelets; hypoxic vaso-occlusion; and direct activation of immune and vascular cells by virus infection. Furthermore, many patients hospitalized with COVID-19 exhibit neutrophil extracellular traps (NETs) in their blood (*15*, *16*), and these inflammatory cell remnants may also contribute to the prothrombotic milieu (*17*–*20*).

Antiphospholipid syndrome is an acquired thrombophilia, affecting at least 1 in 2000 individuals (*21*). Patients form durable autoantibodies to phospholipids and phospholipid-binding proteins (aPL antibodies), such as prothrombin and β_2_ glycoprotein I (β_2_GPI). These autoantibodies engage cell surfaces, where they activate endothelial cells, platelets, and neutrophils (*22*, *23*), thereby tipping the blood-endothelium interface toward thrombosis. A key feature of antiphospholipid syndrome is its ability to promote thrombosis in vascular beds of all sizes, including both arterial and venous circuits. The catastrophic variant of antiphospholipid syndrome is frequently fatal and bears some similarities to the diffuse coagulopathy seen in patients with COVID-19 (*24*). Classification criteria for antiphospholipid syndrome (last updated in 2006) seek persistently positive testing for anticardiolipin autoantibodies (aCL antibodies) or anti–β_2_GPI autoantibodies (aβ_2_GPI antibodies) (*25*). The lupus anticoagulant test (a functional assay that screens for aPL antibodies based on their paradoxical ability to prolong in vitro clotting assays) is also included in the criteria and detects a variety of species of aPL antibodies including anti-phosphatidylserine/prothrombin autoantibodies (aPS/PT antibodies) (*26*).

Reports of aPL antibodies in COVID-19 and their possible relationship to thrombosis have begun to emerge in case reports and case series (*27*–*32*). Whereas viral infections are well-known triggers of transient aPL antibody production (*33*–*36*), the extent to which these short-lived autoantibodies are pathogenic has not been well defined. Here, we aimed to test for several types of aPL antibodies in serum samples from a cohort of 172 patients hospitalized with COVID-19. We also asked whether purified immunoglobulin G (IgG) fractions from these patients had prothrombotic properties in vitro and in two mouse models of thrombosis.

## RESULTS

### Prevalence of aPL antibodies in serum from patients hospitalized with COVID-19

Serum samples from 172 patients hospitalized with COVID-19 (table S1) were evaluated for eight different types of aPL antibodies. Of the 172 patients, 19% died and 8% remained in the hospital at the time of this analysis. Eighty-nine patients tested positive for at least one type of aPL antibody based on the manufacturer’s cutoff, representing 52% of the entire cohort (Table 1). The lupus anticoagulant test, a functional assay that relies on altered coagulation times in plasma to detect antiphospholipid antibody activity, was not performed here given lack of access to fresh plasma samples. Among the various aPL antibodies tested, aPS/PT IgG had the highest prevalence (24%), followed by aCL IgM (23%) and aPS/PT IgM (18%) (Table 1). Forty-one patients (24%) were positive for more than one type of aPL antibody, and 13 (8%) were positive for more than two types of aPL antibody. Fifty-two patients (30%) had at least one moderate- to high-titer aPL antibody (Table 1). Thirty-six patients had serum samples taken at multiple time points available for aPL antibody testing, which enabled longitudinal analysis (fig. S1). In Table 1, the highest available aPL antibody serum titer was used to classify positivity for each of these 36 patients. Seropositivity was also assessed using only the first available serum sample, with similar rates of positivity as presented in Table 1 (table S2). To further elucidate the antigen specificity of autoantibodies in serum samples positive for aPS/PT antibodies, we measured aPS autoantibodies (aPS antibodies) in these serum samples. Neither aPS IgG nor aPS IgM correlated with aPS/PT antibody serum titers, suggesting that aPS/PT antibodies in COVID-19 patient serum primarily recognized prothrombin (fig. S2). In summary, serum samples from 52% of patients hospitalized for COVID-19 were positive for aPL antibodies, with about two-thirds of those being detected at moderate-to-high titers. The majority of positive serum samples were associated with three types of autoantibodies: aPS/PT IgG, aCL IgM, and aPS/PT IgM.

**Table 1:** Prevalence of antiphospholipid antibodies in serum from patients with COVID-19 (*n* = 172). The manufacturer’s cutoff: aCL IgG, IgM, IgA = 20 IgG, IgM, IgA phospholipid units; aβ_2_GPI IgG, IgM, IgA = 20 standard IgG, IgM, IgA units; aPS/PT IgG, IgM = 30 IgG, IgM phosphatidylserine units; aPL antibody, antiphospholipid autoantibodies; aCL, anticardiolipin antibodies; aβ_2_GPI, anti–β_2_ glycoprotein I antibodies; aPS/PT, anti-phosphatidylserine/prothrombin antibodies.

**aPL antibody**	**Number of positive** **(manufacturer’s cutoff)**	**%**	**Number of positive** **(titer ≥40 units)**	**%**
aCL IgG	8	4.7	2	1.2
aCL IgM	39	23	13	7.6
aCL IgA	6	3.5	1	0.58
aβ_2_GPI IgG	5	2.9	3	1.7
aβ_2_GPI IgM	9	5.2	7	4.1
aβ_2_GPI IgA	7	4.1	3	1.7
aPS/PT IgG	42	24	21	12
aPS/PT IgM	31	18	21	12
Any positive aPL	89	52	52	30

### Clinical correlates of aPL antibodies

We next asked whether the presence of aPL antibodies was associated with various clinical characteristics. Specifically, we assessed potential correlations of aPL antibodies with the ratio of oxygen saturation to fraction of inspired oxygen (SpO_2_/FiO_2_, i.e., oxygenation efficiency), C-reactive protein in serum, D-dimer concentrations in plasma, platelet counts, absolute neutrophil counts, calprotectin in serum (a marker of neutrophil activation), and myeloperoxidase (MPO)–DNA complexes in serum (markers of NETs) (Table 2). Titers of aCL IgM correlated with all of these clinical and laboratory variables (Table 2). Neutrophil activation as indicated by calprotectin in serum was most consistently associated with the presence of aPL antibodies (Table 2). We also assessed a previously devised tool called the aPL score, which integrates and prioritizes data from the various aPL antibody types tested (*37*). The aPL score demonstrated a positive correlation with platelet count (*P* = 0.03), neutrophil activation (*P* = 0.0007), and the presence of NETs (*P* = 0.02) (Table 2).

**Table 2:** Correlation of antiphospholipid antibodies with clinical and laboratory variables in patients with COVID-19. Thiry-six patients had serum samples from multiple time points; for those patients, only the first available serum sample was used for determining correlations. ns, not significant; NETs, neutrophil extracellular traps; MPO, myeloperoxidase.

	**aPL score** **(modified)**		**aCL IgG**		**aCL IgM**		**aβ_2_GPI IgG**		**aβ_2_GPI IgM**		**aPS/PT IgG**		**aPS/PT IgM**	
**Spearman**	***r***	***P***	***r***	***P***	***r***	***P***	***r***	***P***	***r***	***P***	***r***	***P***	***r***	***P***
**Clinical and laboratory variables**														
SpO_2_/FiO_2_	−0.051	ns	−0.16	*	−0.19	*	−0.10	ns	−0.022	ns	−0.11	ns	−0.16	*
C-reactive protein	0.031	ns	0.15	ns	0.17	*	0.075	ns	−0.040	ns	0.058	ns	0.16	*
D-dimer	0.087	ns	0.092	ns	0.24	**	0.041	ns	0.000	ns	0.005	ns	0.037	ns
Platelet count	0.17	*	0.095	ns	0.29	****	0.17	*	0.11	ns	−0.009	ns	0.23	**
Neutrophil count	0.10	ns	0.13	ns	0.19	*	0.047	ns	0.041	ns	−0.008	ns	0.096	ns
Calprotectin	0.26	***	0.29	****	0.28	***	0.11	ns	0.090	ns	0.25	***	0.23	**
NETs (MPO/ DNA)	0.18	*	0.16	*	0.25	***	0.20	**	0.13	ns	0.033	ns	0.23	**

*P < 0.05, **P < 0.01, ***P < 0.001, and ****P < 0.0001.

**Fig. 1:**
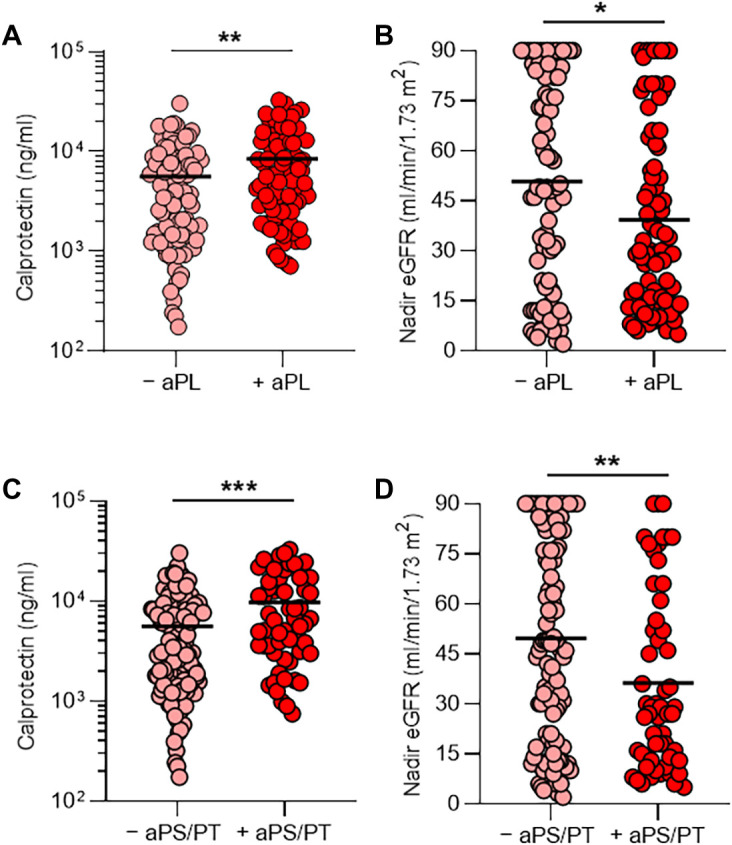
aPL antibodies, NET release, and renal function. Serum samples were obtained from 172 patients hospitalized with COVID-19. (**A** and **B**) Patients were divided into two groups on the basis of whether their serum samples were positive (+) or negative (−) for the presence of aPL antibodies (positivity was based on the manufacturer’s threshold). Shown is the amount of calprotectin in serum, a measure of neutrophil activation (A), and the clinical estimated glomerular filtration rate (eGFR) (B) for the two groups. (**C** and **D**) Patients were divided into two groups on the basis of whether their serum samples were positive (+) or negative (−) for the presence of aPS/PT antibodies (IgG and IgM considered together); the manufacturer’s thresholds were used to determine positivity. Shown is the amount of calprotectin (C) and the eGFR (D) for the two groups. Groups were analyzed by an unpaired *t* test: *P < 0.05, **P < 0.01, and ***P < 0.001. Horizontal black bars represent the mean. For patients who had serum samples available at multiple time points, only the first available serum sample was used in this analysis.

We then examined clinical variables as they related to positive aPL antibody thresholds for each enzyme-linked immunosorbent assay (ELISA) test. A positive test for any aPL antibody was associated with higher calprotectin in serum (*P* = 0.009) and lower clinical estimated glomerular filtration rate (eGFR; *P* = 0.03) (Fig. 1, A and B). These associations were also observed when comparing patient serum samples that were positive for aPS/PT antibodies to serum samples from the remainder of the cohort (calprotectin, *P* = 0.0008; eGFR, *P* = 0.008) (Fig. 1, C and D) or serum samples without aPL antibodies (calprotectin, *P* = 0.001; eGFR, *P* = 0.01) (fig. S3). Nadir eGFR was lower in patients with a history of renal disease compared with those without (*P* = 0.01) (fig. S4). Oxygenation efficiency tended to be impaired in patients with serum samples positive for aPL or aPS/PT antibodies compared with those whose serum samples lacked these antibodies, although group comparisons did not reach statistical significance (fig. S5). Similarly, peak troponin in serum and peak D-dimer in plasma tended to be higher in patients with a positive test for any aPL antibody or anti-PS/PT antibody, respectively (fig. S6). Given that obesity can affect the D-dimer concentration in plasma, we compared D-dimer plasma concentrations in patients with COVID-19 with or without obesity, but did not find a difference (fig. S7). Thus, the presence of aPL antibodies in serum samples from patients with COVID-19 correlated with various clinical characteristics, especially neutrophil activation and impaired renal function.

### IgG isolated from COVID-19 patient serum triggers release of NETs

**Fig. 2:**
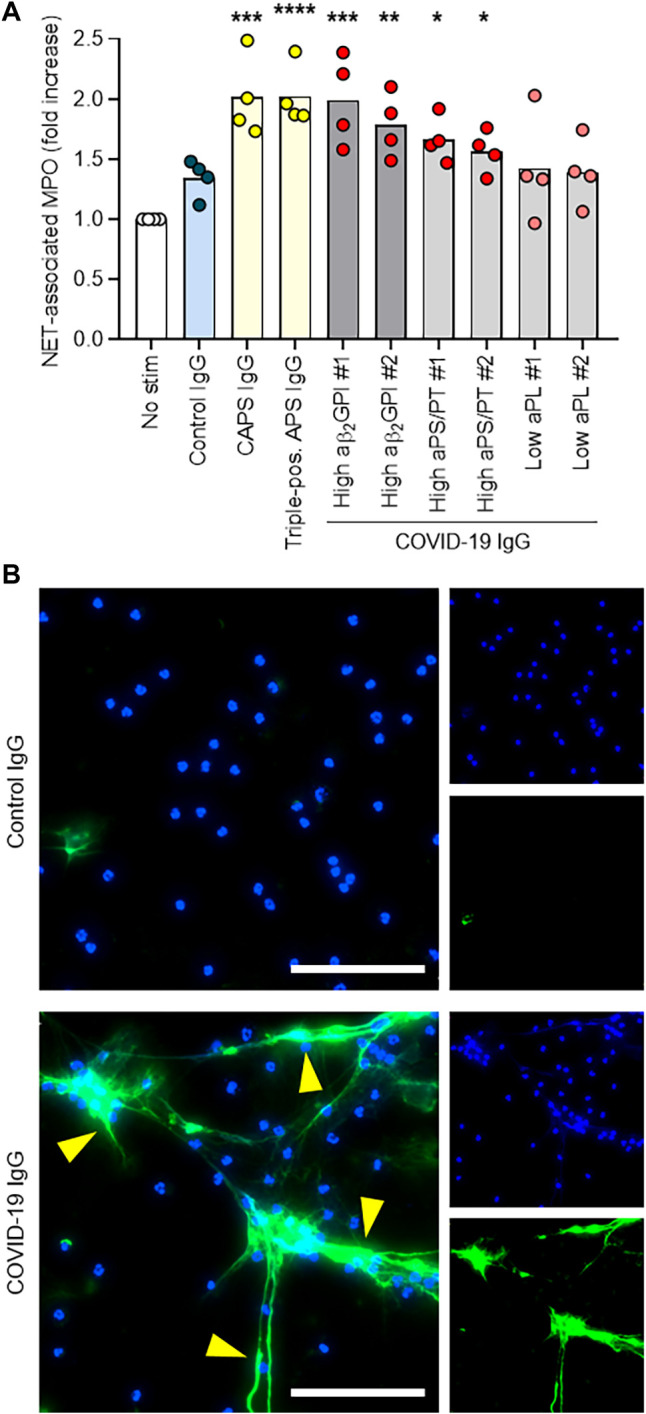
COVID-19 patient IgG promotes NET release from normal neutrophils in vitro. (**A**) Control neutrophils were isolated from healthy individuals and cultured in the presence of human IgG (10 μg/ml) for 3 hours. IgG fractions were obtained from patients with COVID-19 who were or were not positive for aPL antibodies (aPS/PT or aβ_2_GPI as indicated), and from patients with antiphospholipid syndrome (APS) or catastrophic APS (CAPS). NET release was measured by the enzymatic activity of myeloperoxidase (MPO) after solubilization of NETs with micrococcal nuclease; fold increase is plotted relative to unstimulated neutrophils (no stim). Data are derived from four independent experiments. Comparisons were to the unstimulated group by one-way ANOVA with correction for multiple comparisons by Dunnett’s method: *P < 0.05, **P < 0.01, ***P < 0.001. (**B**) Representative images show released NETs, indicated by yellow arrows. DNA, blue; neutrophil elastase, green. Scale bars, 100 μm.

Work by our group and others has revealed that one prothrombotic function of aPL antibodies in patients with antiphospholipid syndrome is to trigger release of NETs (*23*, *38*). Given that we recently detected elevated NETs in serum from patients with COVID-19 (*15*), we reasoned that IgG fractions purified from serum of patients with COVID-19 might be able to trigger NET release. We selected two patients with COVID-19 with high serum aβ_2_GPI IgG, two patients with COVID-19 with high serum aPS/PT IgG, and two patients with COVID-19 who lacked serum aPL antibodies. From these patients, we purified total IgG fractions and tested them alongside IgG pooled from two patients with active catastrophic antiphospholipid syndrome as well as a separate IgG pool from five patients with antiphospholipid syndrome who tested positive for aCL antibodies, aβ_2_GPI antibodies, and lupus anticoagulant. The purity of isolated COVID-19 patient IgG was verified by SDS–polyacrylamide gel electrophoresis (PAGE; fig. S8). To quantify NET release in vitro, we measured MPO activity released into the supernatant after digestion of NET DNA with micrococcal nuclease. The release of NETs from neutrophils isolated from healthy individuals doubled (compared with unstimulated neutrophils) when neutrophils were cultured with COVID-19 patient IgG samples positive for aPL antibodies (Fig. 2A and data file S1). This was similar to the degree of NET release induced in neutrophils by IgG samples from patients with antiphospholipid syndrome (*P* < 0.0001) or catastrophic antiphospholipid syndrome (*P* = 0.0001). Representative images of NET release induced by COVID-19 patient IgG are shown in Fig. 2B. We have previously shown that dipyridamole—an antithrombotic medication—can attenuate aPL antibody–mediated prothrombotic NET release by surface adenosine A_2A_ receptor agonism (*39*). Here, we found that dipyridamole also suppressed COVID-19 patient IgG–mediated NET release from neutrophils in vitro (fig. S9). In summary, IgG fractions purified from COVID-19 patient serum positive for aPL antibodies promoted NET release similar to IgG isolated from individuals with established antiphospholipid syndrome.

### IgG isolated from aPL antibody–positive patient serum potentiates thrombosis in mice

**Fig. 3:**
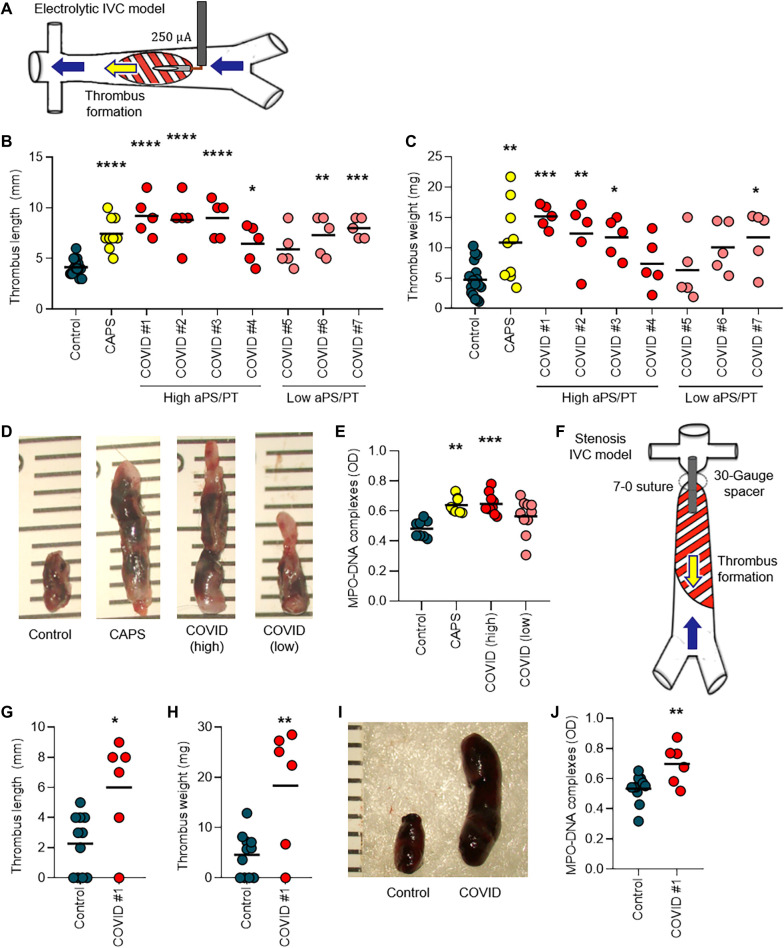
IgG from patients with COVID-19 potentiates thrombosis in mice. (**A**) Schematic shows thrombus initiation in the inferior vena cava (IVC) of mice by local electrolysis leading to free radical generation and activation of the endothelium. (**B** and **C**) Mice were administered IgG from healthy individuals (control), from patients with COVID-19 who had high or low aPS/PT antibodies, or from patients with catastrophic APS (CAPS). Just before intravenous administration of IgG, mice were subjected to local electrolysis in the IVC. Thrombus length (B) and weight (C) were determined 24 hours after IgG injection. Scatter plots with individual data points (each point represents a single mouse) are presented. (**D**) Shown are photographs of representative thrombi from the experiments presented in (B) and (C). The rulers are measuring thrombi in millimeters. (**E**) Serum samples from mice in the experiments presented in (B) and (C) were tested for NET remnants measured by an ELISA that detected myeloperoxidase (MPO)–DNA complexes. Scatter plots with individual data points (each point represents a single mouse) are presented. OD, optical density. (**F**) Schematic shows thrombus initiation in the IVC of mice by a stenosis that was induced via placement of a fixed suture over a spacer that was subsequently removed. (**G** and **H**) Mice were treated intravenously with IgG from a healthy individual (control) or from a patient with COVID-19 with high aPS/PT antibodies. Just before intravenous administration of IgG, stenosis was induced. Twenty-four hours later, thrombus length (G) and weight (H) were determined. Scatter plots with individual data points (each point represents a single mouse) are presented. (**I**) Shown are photographs of representative thrombi from the experiments presented in (G) and (H). (**J**) Serum samples from mice in the experiments presented in (G) and (H) were tested for NET remnants measured by an ELISA that detected MPO-DNA complexes. Scatter plots with individual data points (each point represents a single mouse) are presented. Horizontal black bars represent the mean. Comparisons were by either one-way ANOVA with correction for multiple comparisons by Dunnett’s method (B, C, and E) or unpaired t test (G, H, and J): *P < 0.05, **P < 0.01, ***P < 0.001, and ****P < 0.0001.

We next sought to determine whether IgG fractions from COVID-19 patient serum could accelerate thrombosis. When tested in an in vitro cell-free thrombin generation assay, IgG fractions purified from COVID-19 patient serum did not have demonstrable clot-accelerating activity (fig. S10). Nevertheless, we speculated that a prothrombotic phenotype might still be observed in the cell-enriched vascular environment of mice. We have previously reported that IgG isolated from serum of patients with either triple-positive antiphospholipid syndrome or catastrophic antiphospholipid syndrome accelerates large-vein thrombosis in various mouse models of inferior vena cava thrombosis (*38*–*40*). Here, we asked whether COVID-19 patient serum IgG might behave similarly to enhance thrombosis in these mouse models. We first used a mouse model in which a copper wire was placed inside the inferior vena cava to activate the endothelium by electrolysis-mediated free radical generation (Fig. 3A). In this model, IgG isolated from patients with COVID-19 with a high serum titer of aPS/PT IgG increased thrombus extension (Fig. 3B) and overall accretion (Fig. 3, C and D, and data file S2) 24 hours after IgG intravenous injection. The high aPS/PT serum titer samples also increased NET remnants in mouse serum (*P* = 0.0004), similar to IgG from patients with catastrophic antiphospholipid syndrome (*P* = 0.001) (Fig. 3E and data file S3), and demonstrated a tendency toward higher expression of citrullinated histone H3 (a biochemical marker of NETs) in mouse thrombi by Western blotting (fig. S11). To confirm these findings, we turned our attention to an independent mouse model in which the inferior vena cava was narrowed just distal to the renal vein by a fixed suture placed over a spacer that was subsequently removed (Fig. 3F); thrombus size was measured 24 hours after IgG intravenous injection. In this “stenosis” mouse model of thrombosis, IgG from a patient with COVID-19 with a high aPS/PT serum titer also increased thrombus extension (*P* = 0.01) (Fig. 3G), thrombus accretion (*P* = 0.003) (Fig. 3, H and I, and data file S4), and circulating NET remnants (*P* = 0.008) (Fig. 3J and data file S3) 24 hours after IgG intravenous injection. Together, these data indicate that IgG fractions from some patients with acute COVID-19 were able to accelerate thrombosis in vivo.

## DISCUSSION

Antiphospholipid autoantibodies (aPL antibodies) are a heterogeneous group of antibodies that underlie the pathogenesis of antiphospholipid syndrome via their interactions with phospholipid-binding plasma proteins such as β_2_GPI, prothrombin, thrombomodulin, plasminogen, antithrombin III, protein C, protein S, annexin II, annexin V, and likely others (*22*, *41*–*46*). The association between various infections and the induction of aPL antibodies has long been recognized (*47*–*52*). For example, one study of 100 cases reported in Medline from 1983 to 2003 found the most commonly reported aPL antibody–associated infections to be skin infections (18%), pneumonia (14%), and urinary tract infections (10%); common pathogens included HIV (17%), varicella-zoster virus (15%), and hepatitis C virus (13%) (*50*). Regarding specific aPL antibodies, aCL IgG and IgM (typically lacking anti-β_2_GPI antibody activity) have been most commonly reported (*48*, *52*–*57*). Most of these virus-associated aPL antibodies are thought to be transient (*35*, *54*, *58*). Although the clinical implications of transient virus–associated aPL antibodies remain to be fully defined, a recent review of 163 published cases of virus-associated aPL antibodies found thrombotic events in 116 cases (*35*). Even acknowledging the likelihood of sampling and publication bias, these data (along with the data presented here for individuals with severe COVID-19) suggest that some transient aPL antibodies may still have prothrombotic potential. Whether similar antibodies would be detected in patients with less symptomatic COVID-19 presentation—some of whom do experience thrombotic events—awaits further study.

The most severe presentation of antiphospholipid syndrome is its catastrophic variant, which fortunately affects only a minority of patients with antiphospholipid syndrome, typically at times of stress such as infection, surgery, or withdrawal of anticoagulants (*59*). Catastrophic antiphospholipid syndrome involves derangements of both inflammatory and thrombotic pathways and affects multiple organs in the body simultaneously (*59*). In the largest series of patients with catastrophic antiphospholipid syndrome assembled, the most commonly affected organs were kidneys (73%), lungs (60%), brain (56%), heart (50%), and skin (47%) (*60*). Whereas multiorgan failure certainly complicates severe cases of COVID-19, the lungs are typically the most severely affected organ. We speculate that local immune stimulation due to viral infection (including potentially the infection of endothelial cells) could synergize with circulating aPL antibodies and thereby lead to a particularly severe thrombo-inflammatory insult to the lungs of patients with COVID-19.

Many studies from the general thrombosis literature have revealed that activated neutrophils, and in particular NET formation, contribute to the propagation of thrombi affecting arterial, venous, and microscopic vascular beds (*61*, *62*). NETs have also been recently implicated in the pathogenesis of antiphospholipid syndrome. Our group has reported that serum samples from patients with antiphospholipid syndrome, as well as purified aPL antibodies, trigger neutrophils to release NETs (*23*). The potential in vivo relevance of this observation has been confirmed in mouse models of aPL antibody–mediated large-vein thrombosis in which either depletion of neutrophils or digestion of NETs was protective (*38*). Neutrophils from patients with antiphospholipid syndrome also appear to have increased adhesive potential, which is dependent on the activated form of integrin Mac-1. This proadhesive phenotype amplifies neutrophil-endothelium interactions, potentiates NET formation, and potentially lowers the threshold for thrombosis (*63*). Therapies that target NET formation have the potential to treat thrombotic diseases. For example, selective agonism of the adenosine A_2A_ receptor suppresses aPL antibody–mediated NETosis in a protein kinase A–dependent fashion (*39*). A_2A_ receptor agonism also reduces thrombosis in the inferior vena cava of both control mice and mice treated with aPL antibodies. Dipyridamole, which is known to potentiate adenosine receptor signaling by increasing extracellular concentrations of adenosine and interfering with the breakdown of adenosine 3′,5′-monophosphate (cAMP), also suppresses aPL antibody–mediated NETosis and mitigates venous thrombosis in mice (*64*). A small study from China showed that dipyridamole suppressed D-dimer elevation and improved platelet counts in patients with COVID-19 (*65*). Whereas we have demonstrated here that dipyridamole mitigated NET release mediated by IgG from patients with COVID-19, prospective randomized clinical trials (https://clinicaltrials.gov/ct2/show/NCT04391179) are needed to evaluate clinical outcomes among patients with COVID-19 treated with dipyridamole (*64*).

aPL antibodies are defined on the basis of their inclusion in the updated Sapporo classification criteria: namely, aCL IgG and IgM, aβ_2_GPI IgG and IgM, and lupus anticoagulant (*25*). Of these, lupus anticoagulant is generally accepted as the best indicator of a high-risk aPL antibody profile (*66*–*71*). There are certainly reports of patients with seronegative antiphospholipid syndrome who have classic features of this disease but have tested negative for traditional aPL antibodies (*72*). Some noncriteria aPL antibodies found in the past 20 years have shown promising clinical utility in identifying antiphospholipid syndrome. Among those are aPS/PT IgG and IgM, as well as the IgA isotypes of aCL and aβ_2_GPI antibodies. Retrospective studies have suggested that aβ_2_GPI IgA is associated with thrombosis in patients with lupus [odds ratio (OR), 2.8; 95% confidence interval (CI), 1.3 to 6.2] (*73*). A recent review of 10 retrospective studies (17,75 patients with lupus or primary antiphospholipid syndrome and 628 healthy controls) detected a strong association between aPS/PT antibodies and thrombotic events (OR, 5.11; 95% CI, 4.2 to 6.3) (*74*). Furthermore, serological agreement between aPS/PT IgG and IgM and high-risk aPL antibody profiles—especially the presence of lupus anticoagulant—has been demonstrated in a recent study of 95 well-characterized patients with primary antiphospholipid syndrome (*75*). Whereas the clinical implications of aPS/PT antibodies during viral infection remain to be comprehensively defined, we found here that IgG fractions containing high titers of these antibodies triggered NET release from neutrophils in vitro and accelerated thrombosis in vivo. Notably, IgG purified from patients with COVID-19 with low aPS/PT serum titers demonstrated some activity in potentiating thrombosis (although high aPS/PT serum titer IgG fractions provided a more robust response). It is possible that aPL antibodies are but one species of a broader acute natural antibody response that is prothrombotic in COVID-19 disease.

The orchestration of autoimmunity against phospholipids in COVID-19 is likely a complex interplay between genetic predisposition, historical antigen exposures, and a hyperactivated host immune response in the setting of a unique environmental trigger—infection with SARS-CoV-2 (*76*). It is expected that aPL antibodies of the IgM isotype (which are designed for rapid mobilization) predominate in our COVID-19 patient cohort, where they correlate with markers of neutrophil activation and NET release. The relationship between aPL antibodies and NETs in COVID-19 is potentially bidirectional. NETs are a known source of autoantigens, and cytokines released in parallel with NETosis may also facilitate NET-associated autoantibody propagation (*77*–*80*). An example of a cytokine that could play such a role is B cell–activating factor (BAFF), an important mediator of the maturation of B cells into antibody-producing cells (*81*). For example, neutrophil-derived BAFF likely participates in the production of anti–double-stranded DNA antibodies in lupus (*78*). In COVID-19, it is possible that production of aPL antibodies potentiates NET formation and BAFF release. This may further enhance the survival and differentiation of phospholipid-reactive B cells and, in some cases, class switching to the IgG isotype. The interplay between COVID-19 and humoral immunity is an area that merits further study.

There are several potential clinical implications of these findings. Patients with catastrophic antiphospholipid syndrome are regularly treated with heparin, corticosteroids, and plasmapheresis (with the latter leading to a demonstrable improvement in outcomes) (*82*). Whereas both anticoagulation and corticosteroids have shown some promise to date in treatment of COVID-19, plasmapheresis has not been systematically explored. One wonders whether this could provide benefit in the subgroup of patients with COVID-19 with high titers of aPL antibodies. At the same time, convalescent plasma is receiving increasing attention as an approach to treating severe cases of COVID-19. Defining the extent to which convalescent plasma may contain aPL antibodies or other prothrombotic autoantibodies in addition to protective anti–SARS-CoV-2 antibodies is another potential area for future investigation.

Our study has several limitations. We did not have access to the fresh plasma samples that would be required for lupus anticoagulant testing (which would have provided additional context and risk stratification for the aPL antibody profiling results). We speculate that some of the patients with COVID-19 in our cohort whose serum samples were positive for aPS/PT antibodies would have displayed a lupus anticoagulant phenotype, as reported recently (*26*), but proving that will require further study and prospective access to plasma samples. We were also not able to define a clear link between circulating aPL antibodies and large artery/vein thrombosis in our patient cohort. Eleven patients in our cohort had thrombotic events, and 55% of them were positive for aPL antibodies. Notably, aggressive anticoagulation has been regularly used at our institution in the context of COVID-19, and many patients with COVID-19 have been treated prophylactically with therapeutic doses of anticoagulants. It should also be noted that aPL antibodies were not tested on a defined day of hospitalization, but rather when a serum sample became available to the research laboratory. Future studies should endeavor to systematically track aPL antibodies over the full course of hospitalization of patients with COVID-19, and perhaps especially at and after the time of discharge.

As we await definitive antiviral and immunological solutions to the current COVID-19 pandemic, we posit that testing for aPL antibodies, including aPS/PT antibodies, may lead to improved risk stratification and personalization of treatment for patients with COVID-19. We also suggest further investigation of aPL antibodies as a contributor to the complex thrombo-inflammatory milieu of COVID-19.

## MATERIALS AND METHODS

### Study design

In this cross-sectional cohort study of 172 patients hospitalized with COVID-19, we aimed to measure subtypes of aPL antibodies in serum samples from these patients. We also asked whether purified IgG fractions from patients positive for serum aPL antibodies had prothrombotic properties in NET release assays in vitro and in two mouse models of venous thrombosis in vivo. In studies of the two mouse models of inferior vena cava thrombosis (the electrolysis and stenosis models), investigators doing the surgeries were blinded to the experimental conditions. No data points were excluded as outliers from either the human or mouse studies.

Our human cohort study complied with all relevant ethical regulations and was approved by the University of Michigan Institutional Review Board (IRB; HUM00179409), which waived the requirement for informed consent given the discarded nature of the serum samples.

Mice were housed in a specific pathogen–free barrier facility and fed standard chow. Experimental protocols were approved by the University of Michigan Institutional Animal Care and Use Committee (PRO00008113), and all relevant ethical regulations were followed. Male C57BL/6 mice were purchased from the Jackson laboratory and used for experiments at 10 to 12 weeks of age.

### Serum samples from patients with COVID-19

Serum samples from 172 patients hospitalized with COVID-19 were used in this study (table S1). Blood was collected into serum separator tubes containing clot activator and serum separator gel by a trained hospital phlebotomist. After completion of biochemical testing ordered by the clinician, the remaining serum was stored for clinical testing at 4°C for up to 48 hours before release to the research laboratory. Serum samples were immediately divided into small aliquots and stored at −80°C until the time of testing. All 172 patients had a confirmed COVID-19 diagnosis based on a U.S. Food and Drug Administration (FDA)–approved RNA testing. Fifty of these 172 patients were included in our prior study evaluating the role of NETs in COVID-19 (*15*). All patients were also included in our prior study evaluating the role of calprotectin in COVID-19 (*16*). However, aPL antibodies were not considered in either study (*15*, *16*).

### Quantification of aPL antibodies

aPL antibodies were quantified in sera using Quanta Lite ACA IgG, ACA IgM, ACA IgA, β_2_GPI IgG, β_2_GPI IgM, β_2_GPI IgA, aPS IgG, aPS IgM, aPS/PT IgG, and aPS/PT IgM kits (Inova Diagnostics Inc.) according to the manufacturer’s instructions. All assays are approved for clinical use and received 510(k) clearance from the FDA. Quanta Lite aPL antibody ELISAs (Inova Diagnostics) are well recognized by the international antiphospholipid syndrome research community and are used by the largest international antiphospholipid syndrome clinical research network registry, APS ACTION, in its core laboratories as the “gold standard” for aPL antibody testing (*83*, *84*). Here, IgG, IgM, and IgA aCL antibody assays were reported in GPL (IgG PhosphoLipid units), MPL (IgM PhosphoLipid units), APL (IgA PhosphoLipid units), respectively; aβ_2_GPI antibody assays were reported in SGU (Standard IgG Units), SMU (Standard IgM Units), SAU (Standard IgA Units); aPS assay were reported in GPS (IgG PhosphatidylSerine unit), MPS (IgM PhosphatidylSerine unit); and aPS/PT antibody assays were reported in IgG and IgM units, all per the manufacturer’s specifications. These various units are in accordance with the international consensus guidelines on aPL antibody testing from the 13th International Congress on Antiphospholipid Antibodies (*85*). Per the manufacturer, the establishment of cutoff values for all Quanta Lite aPL antibody assays is based on balancing sensitivity and specificity to achieve optimal clinical utility. For example, in the case of aPS/PT IgG/IgM (per the manufacturer’s documentation), a total of 91 patients with antiphospholipid syndrome, 247 healthy controls, and 43 diseased controls were tested. The threshold chosen resulted in a specificity of 99% for aPS/PT IgG and 98.7% for aPS/PT IgM. A previously described aPL score was used to integrate summarize aPL antibodies profiles, with some adaptations (*37*). Here, aPL score was calculated for each patient by adding points corresponding to the different types and titers of aPL antibodies, weighted as below: high-titer aCL IgG (≥40 GPL) = 20 points; low-titer aCL IgG (≥20 GPL) = 4 points; aCL IgM (≥20 MPL) = 2 points; high-titer aβ_2_PGI IgG (≥40 SGU) = 20 points; low-titer aβ_2_PGI IgG (≥20 SGU) = 6 points; aβ_2_PGI IgM (≥20 SMU) = 1 point; high-titer aPS/PT IgG (≥40 units) = 20 points; low-titer aPS/PT IgG (≥30 units) = 13 points; and aPS/PT IgM (≥30 units) = 8 points.

### Quantification of S100A8/A9 (calprotectin)

Calprotectin was measured with the Human S100A8/S100A9 Heterodimer DuoSet ELISA (DY8226-05, R&D Systems) according to the manufacturer’s instructions.

### Quantification of MPO-DNA complexes

MPO-DNA complexes were quantified similarly to what has been previously described (*86*). This protocol used several reagents from the Cell Death Detection ELISA kit (Roche). First, a high-binding EIA/RIA 96-well plate (Costar) was coated overnight at 4°C with anti-human MPO antibody (Bio-Rad 0400-0002), diluted to a concentration of 1 μg/ml in coating buffer (Cell Death kit). The plate was washed two times with wash buffer [0.05% Tween 20 in phosphate-buffered saline (PBS)], and then blocked with 4% bovine serum albumin in PBS (supplemented with 0.05% Tween 20) for 2 hours at room temperature. The plate was again washed five times, before incubating for 90 min at room temperature with 10% serum or plasma in the aforementioned blocking buffer (without Tween 20). The plate was washed five times and then incubated for 90 min at room temperature with 10× anti-DNA antibody [horseradish peroxidase (HRP) conjugated; from the Cell Death kit] diluted 1:100 in blocking buffer. After five more washes, the plate was developed with 3,3′,5,5′-tetramethylbenzidine (TMB) substrate (Invitrogen) followed by a 2N sulfuric acid stop solution. Absorbance was measured at a wavelength of 450 nm using a Cytation 5 Cell Imaging Multi-Mode Reader (BioTek). Data were normalized to in vitro–prepared NET standards included on every plate, which were quantified on the basis of their DNA content.

### Purification of human IgG fractions

IgG was purified from COVID-19, APS, or control sera with a Protein G Agarose Kit following the manufacturer’s instructions (Pierce). Briefly, serum was diluted in IgG binding buffer and passed through a Protein G Agarose column at least five times. IgG was then eluted with 0.1 M glycine and then neutralized with 1 M tris. This was followed by overnight dialysis against PBS at 4°C. IgG purity was verified with Coomassie staining, and concentrations were determined by bicinchoninic acid (BCA) protein assay (Pierce) according to the manufacturer’s instructions. All IgG samples were determined to have an endotoxin level below 0.1 EU/ml by the Pierce LAL Chromogenic Endotoxin Quantitation Kit (A39552) according to the manufacturer’s instructions. This kit offers high sensitivity with a linear detection range of 0.01 to 1.0 EU/ml.

### Human neutrophil purification and NETosis assay

Collection of healthy human blood was approved by the University of Michigan IRB (HUM00044257). For neutrophil preparation, blood from healthy volunteers was collected into heparin tubes by standard phlebotomy techniques. The anticoagulated blood was then fractionated by density gradient centrifugation using Ficoll-Paque Plus (GE Healthcare). Neutrophils were further purified by dextran sedimentation of the red blood cell layer, before lysing residual red blood cells with 0.2% sodium chloride. Neutrophil preparations were at least 95% pure as confirmed by both flow cytometry and nuclear morphology. To assess NETosis, complementary approaches were used. For the NET-associated MPO assay, neutrophils were resuspended in RPMI media (Gibco) supplemented with 0.5% bovine serum albumin (Sigma-Aldrich) and 0.5% fetal bovine serum (Gibco), which had been heat inactivated at 56°C. Neutrophils (1 × 10^5^ per well) were then incubated in 96-well plates with human IgG (10 μg/ml) for 3 hours. To collect NET-associated MPO, the culture media was discarded (to remove any soluble MPO) and replaced with 100 μl of RPMI supplemented with micrococcal nuclease (5 U/ml; Thermo Fisher Scientific). After 10 min at 37°C, digestion of NETs was stopped with 10 mM EDTA. Supernatants were transferred to a v-shaped 96-well plate and centrifuged at 350*g* for 5 min to remove debris. Supernatants were then transferred into a new plate. To measure MPO activity, an equal volume of TMB substrate (1 mg ml^−1^; Thermo Fisher Scientific) was added to each well. After 10 min of incubation in the dark, the reaction was stopped by the addition of 50 μl of 1 mM sulfuric acid. Absorbance was measured at 450 nm using a Cytation 5 Cell Imaging Multi-Mode Reader. For immunofluorescence microscopy, 1.5 × 10^5^ neutrophils were seeded onto coverslips coated with 0.001% poly-l-lysine (Sigma-Aldrich) and fixed with 4% paraformaldehyde. In some experiments, cells were then permeabilized with 0.1% Triton X-100 for 15 min at room temperature. Blocking was with 1% bovine serum albumin. The primary antibody was against neutrophil elastase (Abcam 21595, diluted 1:100), and the fluorescein isothiocyanate–conjugated secondary antibody was from SouthernBiotech (4052-02, diluted 1:250). DNA was stained with Hoechst 33342 (Invitrogen). Images were collected with a Cytation 5 Cell Imaging Multi-Mode Reader.

### Mouse models of venous thrombosis

To model large-vein thrombosis, we used procedures that we have used previously (*38*, *40*, *87*). For the stenosis model, a laparotomy was performed under anesthesia. After exposure of the inferior vena cava, any lateral branches were ligated using 7-0 Prolene suture (back branches remained patent). A ligature was then fastened around the inferior vena cava over a blunted 30-gauge needle (which served as a spacer). After removal of the spacer, the abdomen was closed. Before recovery from anesthesia, mice received a single intravenous injection of human IgG (500 μg). Twenty-four hours later, mice were humanely euthanized, blood was collected, and thrombus characteristics were measured. The electrolytic model was performed as described (*88*). Briefly, after exposure of the inferior vena cava, any lateral branches were ligated using 7-0 Prolene suture (back branches remained patent). A 30-gauge silver-coated copper wire (KY-30-1-GRN, Electrospec) with exposed copper wire at the end was placed inside a 25-gauge needle, which was inserted into the inferior vena cava and positioned against the anterior wall (where it functioned as the anode). Another needle was implanted subcutaneously, completing the circuit (cathode). A constant current of 250 μA was applied for 15 min. The current was supplied by the voltage-to-current converter that is described in detail in the reference (*88*). After removal of the needle, the abdomen was closed. Before recovery from anesthesia, mice received a single intravenous injection of human IgG (500 μg). Twenty-four hours later, mice were humanely euthanized, blood was collected, and thrombus characteristics were measured.

### Western blotting

Thrombi were homogenized in RIPA buffer with Roche protease inhibitor cocktail pellet and 1% SDS. Protein was quantified using the BCA protein assay kit (Pierce). Thirty micrograms of protein was resolved by SDS-PAGE and then transferred to a polyvinylidene difluoride membrane. Nonspecific binding was blocked with 4% nonfat milk, followed by incubation with primary antibody directed against citrullinated histone H3 (Abcam 5103). Detection was with an HRP-labeled anti-rabbit secondary antibody and an HRP-labeled β-actin antibody, followed by detection using chemiluminescence.

### Thrombin generation assay

Thrombin generation assays were performed using a previously described method (*89*).

### Statistical analysis

Normally distributed data were analyzed by two-sided *t* test, and skewed data were analyzed by Mann-Whitney test. Comparisons of more than two groups were analyzed by one-way analysis of variance (ANOVA) with correction for multiple comparisons by Dunnett’s method. Data analysis was with GraphPad Prism software version 8. Correlations were tested by Spearman’s correlation coefficient. Statistical significance was defined as *P* < 0.05 unless stated otherwise.

## SUPPLEMENTARY MATERIALS

stm.sciencemag.org/cgi/content/full/scitranslmed.abd3876/DC1 Table S1. Demographic and clinical characteristics of the COVID-19 patient cohort. Table S2. Prevalence of aPL antibodies in serum from patients with COVID-19 (*n* = 172) based on the first available sample. Fig. S1. Testing for various aPL antibodies according to day of hospitalization. Fig. S2. Lack of association between aPS/PT antibodies and aPS antibodies in serum from patients with COVID-19. Fig. S3. A positive aPL antibody test is associated with greater neutrophil activation and worse kidney function. Fig. S4. Nadir eGFR in patients with COVID-19 with or without a prior history of renal disease. Fig. S5. aPL antibody status as a predictor of oxygenation efficiency. Fig. S6. aPL antibody status as a predictor of peak troponin and D-dimer. Fig. S7. D-dimer plasma concentrations in patients with or without obesity. Fig. S8. Determination of purity of IgG fractions from patients with COVID-19. Fig. S9. Dipyridamole suppresses IgG-induced NET release. Fig. S10. IgG from patients with COVID-19 does not potentiate thrombin generation in cell-free pooled normal plasma. Fig. S11. Measurement of citrullinated histone H3 in mouse thrombi. Data file S1. NET release measured by MPO activity. Data file S2. Thrombus length and weight in mice. Data file S3. MPO-DNA in mice (stenosis and electrolysis models). Data file S4. Thrombus length and weight in stenosis mouse model.

## Appendix

View/request a protocol for this paper from *Bio-protocol*.
